# Predictors of Risk and Resilience for Posttraumatic Stress Disorder Among Ground Combat Marines: Methods of the Marine Resiliency Study

**DOI:** 10.5888/pcd9.110134

**Published:** 2012-05-10

**Authors:** Dewleen G. Baker, William P. Nash, Brett T. Litz, Mark A. Geyer, Victoria B. Risbrough, Caroline M. Nievergelt, Daniel T. O’Connor, Gerald E. Larson, Nicholas J. Schork, Jennifer J. Vasterling, Paul S. Hammer, Jennifer A. Webb-Murphy, the MRS Team

**Affiliations:** Author Affiliations: William P. Nash, Caroline M. Nievergelt, University of California-San Diego, La Jolla, California; Brett T. Litz, Jennifer J. Vasterling, Veterans Affairs Boston Healthcare System, Boston, Massachusetts; Mark A. Geyer, Victoria B. Risbrough, Daniel T. O’Connor, University of California-San Diego, San Diego, California; Gerald E. Larson, Naval Health Research Center, San Diego, California; Nicholas J. Schork, Scripps Translational Science Institute, La Jolla, California; Paul S. Hammer, Defense Centers of Excellence for Stress and Mental Health and Traumatic Brain Injury, Arlington, Virginia; Jennifer A. Webb-Murphy, Naval Center Combat and Operational Stress Control, San Diego, California.

## Abstract

The Marine Resiliency Study (MRS) is a prospective study of factors predictive of posttraumatic stress disorder (PTSD) among approximately 2,600 Marines in 4 battalions deployed to Iraq or Afghanistan. We describe the MRS design and predeployment participant characteristics. Starting in 2008, our research team conducted structured clinical interviews on Marine bases and collected data 4 times: at predeployment and at 1 week, 3 months, and 6 months postdeployment. Integrated with these data are medical and career histories from the Career History Archival Medical and Personnel System (CHAMPS) database. The CHAMPS database showed that 7.4% of the Marines enrolled in MRS had at least 1 mental health diagnosis. Of enrolled Marines, approximately half (51.3%) had prior deployments. We found a moderate positive relationship between deployment history and PTSD prevalence in these baseline data.

## Introduction

Chronic psychiatric illness such as posttraumatic stress disorder (PTSD) is a major public health problem among current and former military service members, especially those who have served in combat. The prevalence of PTSD among service members and veterans varies widely, but deployment to a war zone is consistently associated with an increased risk for PTSD by a factor of 1.5 to 3.5 across war eras (1). The Iraq and Afghanistan conflicts are no exception (2,3). Additionally, blast-related brain injuries, which are frequently associated with PTSD, are common (3,4). Although suicide rates among active duty personnel have risen since these conflicts started in 2003, reasons for the increase are not fully understood and are being investigated (5). PTSD and mild traumatic brain injury (TBI) appear to be risk factors for suicidal behavior (6). The number of veterans of the current conflicts seeking care at Veterans Health Administration (VHA) facilities has increased (7). Many of these veterans have met screening or diagnostic criteria for PTSD (20%–39%), often co-occurring with depression, anxiety, substance use disorders, and chronic pain (7,8). Associated long-term personal and societal costs are high.

Evidence-based therapies for PTSD have shown only modest efficacy in targeting war trauma (9). Increasingly, military resources are being invested in preventing PTSD. However, scientific advances in understanding the etiology and natural history of PTSD needed to develop effective prevention and treatments have been hampered by reliance on retrospective, cross-sectional research (10). Several prospective investigations of military cohorts have now been initiated (2,3,11). The Marine Resiliency Study (MRS) is singular among these investigations in its combined study of operational units and its biological, psychological, and social scope.

The objective of this article is to describe the research methods used in the MRS, a unique collaboration between the Marine Corps, Navy, Veterans Affairs (VA) Health Services Research and Development (HSR&D), and academia. The description of participant characteristics before deployment combined with future longitudinal data analysis may allow researchers to identify modifiable multisystem risk and resilience factors for combat-related PTSD. The potential factors under investigation are measures of arousal, cardiovascular and physical fitness, mental health, stress reactivity, genetics, neurocognitive function, deployment stressors, and social and military support.

## Methods

### Study design

The MRS entails prospective longitudinal evaluations of biological, psychophysiological, psychosocial, and neurocognitive moderators and mediators of combat stress in Marines recruited from 4 infantry battalions of the 1st Marine Division stationed at Marine Corps Air-Ground Combat Center, 29 Palms, or Camp Pendleton, both in southern California. Commanders of battalions deploying at time frames acceptable to MRS were briefed on study goals, and Marines in available battalions were invited to participate. Testing began on the first of the 4 enrolling battalions in July 2008 and will continue through May 2012. The institutional review boards of the University of California San Diego, VA San Diego Research Service, and Naval Health Research Center approved the study.

The primary study hypothesis is that mental health progression and outcomes among Marines exposed to combat and operational stress, trauma, and loss will be determined by risk and resilience factors across all study domains. Data analysis and hypothesis testing will be iterative, initially testing specific hypotheses within domains, followed by integrated analysis across domains to test the primary study hypothesis. The main goal is to provide the Marine Corps with targets for future prevention interventions. A secondary goal is to enhance scientific understanding of the nature and causes of PTSD.

### Data collection plan

**Figure 1:**
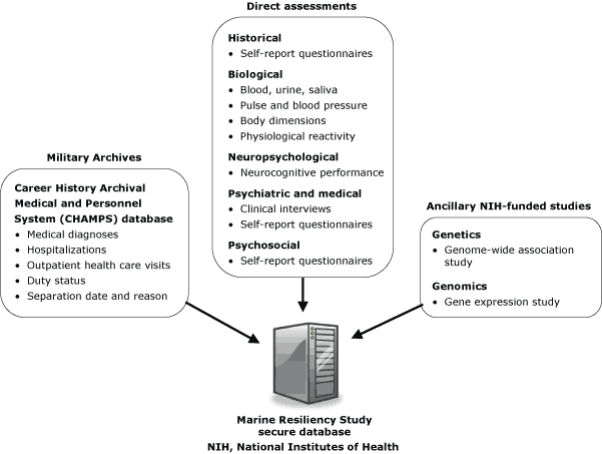
Data sources available to the Marine Resiliency Study.

Close collaboration with the Marine Corps and the Navy, which provides health support for the Marine Corps, enables comprehensive on-site data collection. Data sources include the following: 1) on-site assessments, described in Measures; 2) archival medical record and service data; and 3) ancillary genetic and genomic studies funded by the National Institute of Mental Health (Figure 1). The subject-specific archival data from CHAMPS are integrated with directly collected MRS data and stored in a database maintained at the VA San Diego Medical Center. When the ongoing National Institutes of Health–funded studies of genome-wide association and gene expression are completed, their results will be combined with the MRS database for analysis.

### Study setting and participant recruitment

The MRS on-site assessment plan provides for data collection across each battalion’s 14-month deployment cycle. Marines are evaluated at 4 points relative to their deployments to either Iraq or Afghanistan: T1, approximately 1 month before a 7-month war-zone deployment; T2, 1 week postdeployment; T3, 3 months postdeployment; and T4, 6 months postdeployment. Most assessments are conducted in Marine training spaces on a Marine Corps base. Some participants are assessed at the VA San Diego Medical Center or elsewhere if they have left their military units before study completion for reasons such as relocation, discharge, or injuries during deployment. Special efforts are made to gain access to ill and injured Marines. Individual informed consent is obtained before enrollment at time T1 both for direct assessment and the use of collateral data such as military health and service records. Although the study is sponsored by Marine leadership, participation at each point is voluntary.

### Outcome measures

#### Self-report questionnaires

Participants complete self-report questionnaires (Table 1) in classrooms or other shared spaces furnished with desks or chairs. Many measures selected for use in MRS are identical to those recommended by the federal interagency working group jointly chartered by the VHA, Department of Defense, and National Institutes of Health to recommend common data elements for operational stress research and surveillance (12). Other forms, such as demographic and family history questionnaires tailored for various points, were derived ad hoc for the study. An 18-item Cohesion Scale was created by combining items from 3 validated military social support scales. In addition, we generated a 14-item Inner Conflict Scale, which assesses self-reported acts of omission or commission that may produce inner conflict because they betray deeply held beliefs, a source of psychological injury (13). Several other self-report measures were modified slightly for use in the study, including linking the widely used PTSD Checklist, Peritraumatic Dissociative Experiences Questionnaire, and Life Events Checklist to the single worst or most distressing event identified by the subject during clinical interview. A 34-item Childhood Trauma Questionnaire (CTQ) is modified from the standard 28-item report (14).

**Table 1:** Measures Included in Self-Report Questionnaire Packets at 4 Data Collection Points for Participants in the Marine Resiliency Study

Category	Measure	T1	T2	T3	T4
Personal history	Demographics^a^	X	X	X	X
	Deployment history^a^	X	X	—	—
	Family history^a^	X	—	—	—
	Child Trauma Questionnaire (CTQ)	X	—	—	—
	Life Events Checklist (LEC)	X	X	X	—
	Caffeine use^a^	X	X	X	X
	Tobacco use^a^	X	X	X	X
Personality, coping, and cognition	Connor-Davidson Resilience Scale (CD-RISC)^b^	X	X	X	X
	Response to Stressful Experiences Scale (RSES)	X	X	X	X
	Brief COPE^b^	X	X	X	X
	Positive and Negative Affect Schedule (PANAS)^b^	X	X	X	X
	Dissociative Experiences Scale (DES)^b^	X	—	—	X
	Janoff-Bulman World Assumptions Scale (WAS)^b,c^	X	X	X	—
Psychiatric symptoms	PTSD Checklist (PCL)^c^	X	X	X	X
	Peritraumatic Dissociative Experiences Questionnaire (PDEQ)^b^	X	X	X	X
	Beck Depression Inventory, Revised (BDI-II)^b^	X	X	X	X
	Beck Anxiety Inventory (BAI)^b^	X	X	X	X
	Alcohol Use Disorders Identification Test (AUDIT)^b,c^	X	X	X	X
	Drug Abuse Screening Test (DAST)^b,c^	X	X	X	X
Health and well-being	Short-Form Health Survey, 12-item version (SF-12)	X	X	X	X
	World Health Organization Disability Assessment Schedule (WHO-DAS)	X	X	X	X
Social support	DRRI Deployment Social Support^b^	X	X	X	X
	DRRI Predeployment Social Support^b^	X	X	X	X
	Cohesion Scale^a^	X	X	X	X
	Interpersonal Support Evaluation List (ISEL)^c^	X	X	X	X
Deployment stressors	DRRI Combat Experiences^b^	—	X	—	X
	DRRI Perceived Threat^b^	—	X	—	—
	DRRI Aftermath of Battle^b^	—	X	—	—
	DRRI Difficult Living and Working Environments^b^	—	X	—	—
	DRRI Concerns About Life and Family Disruptions^b^	—	X	—	—
	Inner Conflict Scale (ICS)^a^	—	X	X	—
Health care	Health care utilization^a^	—	X	X	X

Abbreviations and symbols: X, included in packet; —, not included in packet; T1, 1 month before 7-month deployment; T2, 1 week postdeployment; T3, 3 months postdeployment; T4, 6 months postdeployment; PTSD, posttraumatic stress disorder; DRRI, Deployment Risk and Resilience Inventory.

^a^ Created ad hoc for the study and slightly modified between time points to reflect changes.

^b^ For references, see report of federal interagency operational stress common data elements working group (12).

^c^ Slightly modified for the study (see text).

Self-report measures analyzed for this article are Your Health and Well-Being version 2 (SF-12), a measure of functional health, and the CTQ, a measure of childhood adversity (14,15). Age-adjusted norms are available for the SF-12; low SF-12 scores may indicate a risk for PTSD (16). The CTQ is a measure of pre-enlistment stress or adversity.

### Clinical interviews

We interviewed each subject in a sound-dampened private office at points T1, T3, and T4, primarily to assess PTSD symptoms. No clinical interviews were conducted at T2, immediately postdeployment, to minimize subject burden. The primary outcome variable is the Clinician-Administered PTSD scale, a gold-standard structured interview (17). Clinical evaluators also assess panic disorder using a module from the Mini-International Neuropsychiatric Interview, and a history of TBI events using criteria established by the VHA and Department of Defense. TBI symptoms assessed include loss of consciousness (LOC), duration of unconsciousness, and altered mental state (AMS) (eg, confusion or dazed feeling or posttraumatic amnesia). To ensure interrater reliability of structured interviews, all interviewers were trained and certified before each battalion was enrolled, and interrater reliability was assessed on 5% of all interviews for each data collection, in real time with 2 certified raters: 1 rater to conduct the interview and the other rater to provide an observational interrater reliability co-rating.

### Laboratory specimen collection

Autonomic and metabolic traits co-vary with PTSD pathophysiology; we chose stress system, immune and metabolic biomarkers and modulators, C-reactive protein, neuropeptide-Y, and chromogranin-A from plasma; cortisol, cotinine, and α-amylase from saliva; and catecholamines, epinephrine, and norepinephrine from urine to assess these traits (18). Blood, urine, and saliva are collected from each subject at T1, T3, and T4.

#### Body measurements

Height, weight, and waist circumference are measured at T1, T3, and T4, and body mass index is calculated.

#### Hemodynamics

Resting blood pressure and heart rate are measured 3 times, each separated by 3-minute rest periods using the noninvasive DynaPulse oscillometric brachial cuff (PulseMetric, Vista, California), which enables calculation of the hemodynamic parameters of cardiac output, vascular resistance, and vascular compliance (19,20).

#### Physiological reactivity

Modulation of acoustic startle reactivity and heart rate are measured with a battery of 3 tests. Before testing, each participant is screened for hearing impairment and fitted with headphones while seated in a comfortable chair facing a computer monitor. After electrode placement and verification, the participant undergoes the following startle tests: 1) assessment of startle threshold using acoustic tones, 2) test of modulation of acoustic startle response while viewing emotional images or when anticipating image presentation, and 3) test for pre-pulse inhibition and startle habituation (21). Continuous heart rate is recorded throughout testing.

#### Neuropsychological performance

We used a laptop computer running Automated Neuropsychological Assessment Metrics to test each participant’s performance on 2 neurocognitive tasks shown in previous work to be sensitive to deployment (22) and of theoretical relevance to stress: the Continuous Performance Test, a measure of sustained attentional vigilance, and Simple Reaction Time throughput, a measure of reaction time efficiency.

#### Military archives

Participants’ authorization through the Health Insurance Portability and Accountability Act of 1996 allows access to their medical and career history data from the CHAMPS database. Information includes demographic data, medical diagnoses, clinic visits, hospitalizations, duty status, and separation date and reason. For this report, *International Classification of Diseases, Clinical Modification* (ICD-9-CM) mental disorder diagnoses assessed during hospitalizations and ambulatory care were extracted for each subject for the time between enlistment and the participating battalion’s deployment date. For each participant, only the first diagnostic code was used for analysis.

### Study setting and participant recruitment

Subjects were recruited from First Marine Division infantry battalions preparing to deploy from bases in southern California to either Iraq (battalions 1 and 2, 2008) or Afghanistan (battalions 3 and 4, 2009–2010). All active duty members of these operational units were eligible. There were no exclusion criteria.

**Figure 2:**
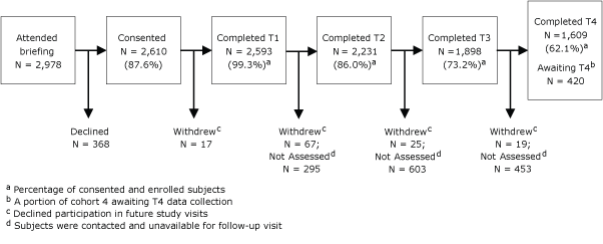
Subject recruitment and retention for the Marine Resiliency Study (N = 2,610) through September 2011.

Participation was offered to 2,978 battalion members, both Marines and accompanying sailors (primarily corpsmen) (Figure 2). Of these battalion members, 2,610 (87.6%) consented to participate and 368 (12.4%) declined. The final battalion is scheduled to complete remaining assessments by May 2012. Dropout rates were highest immediately after deployment (T2) and at the final, 6-month postdeployment data collection (T4). These are the points at which the greatest flux occurred in unit composition. As of January 2012, 20 enrolled participants have been killed in action or died of combat wounds.

### Statistical methods

Analyses were performed in SPSS version 19 (SPSS, Inc, Chicago, Illinois) and included Pearson χ^2^ tests and analysis of variance for between-battalion comparisons and phi (φ) and partial eta squared (ηp^2^), respectively, to estimate effect sizes. CTQ values were linearly transformed for analyses. To test the effects of prior deployments on self-reported health and wellness, we controlled for the potentially confounding effects of age. All comparisons between previously deployed and never-deployed participants were age-adjusted, and group means and standard deviations (SDs) were reported, if appropriate.

## Results

We report demographic, descriptive, and self-report (SF-12, CTQ, and TBI) for all Marines who completed predeployment assessments (Table 2). For all Marines enrolled in MRS battalions at predeployment, the mean (SD) physical health component (PHC) SF-12 score was 53.7 (6.16) and mental health component (MHC) SF-12 score was 50.2 (8.09).

**Table 2:** Baseline Characteristics of Participants in the Marine Resiliency Study ^a^

Characteristic	Battalion 1	Battalion 2	Battalion 3	Battalion 4	Total

	n = 315	n = 721	n = 671	n = 886	n = 2,593
**Age, y^b^**					
Mean (SD)	21.4 (3.1)	21.9 (3.4)	22.7 (3.5)	22.7 (3.7)	22.3 (3.5)
Range	18–42	18–47	18–47	18–43	18–47
**Marital status, %^b^**					
Not married	71.7	68.0	57.7	54.4	61.1
Married	26.3	28.6	38.7	40.4	35.0
Divorced/separated	1.9	3.2	3.6	4.2	3.5
Deployments, %^b^					
Never deployed	46.0	44.5	53.9	48.1	48.4
Previously deployed	54.0	55.3	46.1	51.1	51.3
**Rank, %^b^**					
E1-E3	75.9	75.5	68.9	58.5	68.0
E4-E9	21.0	22.6	27.4	38.4	29.0
O1-O9	3.2	1.7	3.1	2.3	2.4
**Race, %**					
European American	84.4	87.4	78.5	81.6	82.8
African American	2.9	3.7	5.5	5.5	4.7
Asian	3.5	2.5	2.4	2.8	2.7
American Indian	0.6	1.4	1.6	1.5	1.4
Pacific Islander	1.6	1.2	0.9	2.0	1.5
Mixed/other	7.0	3.5	5.8	5.1	5.1
Ethnicity, %					
Not Hispanic	77.8	79.9	73.8	74.7	76.3
Hispanic/Latino	22.2	19.7	25.2	24.7	23.1
**Childhood trauma**					
Total score, mean (SD)	40.8 (13.1)	39.4 (13.4)	38.5 (12.5)	42.0 (14.9)	40.3 (13.7)
Total score, range	25–95.5	25–106.5	25–105.3	25–103.3	25–106.5
Head injury, %	67.9	65.7	58.4	55.1	60.5
TBI with LOC and AMS^c^	54.2	60.5	54.7	56.4	56.9
**Duration of LOC, %^d^**					
≤15 min	63.8	62.6	60.3	59.0	61.1
16–30 min	12.9	14.0	18.7	16.5	15.7
≥30 min	10.3	14.0	11.2	10.6	11.8
Unknown	12.9	9.4	9.8	13.9	11.4
**SF-12 – NEMC mean, (SD)**					
Physical	54.9 (5.7)	53.5 (6.3)	54.0 (6.0)	53.3 (6.2)	53.7 (6.2)
Mental	49.0 (8.4)	49.3 (8.3)	50.7 (7.6)	50.7 (7.8)	50.2 (8.1)

Abbreviations: SD, standard deviation; E1-E9, enlisted; O1-O9, officer; TBI, traumatic brain injury; LOC, loss of consciousness; AMS, altered mental state; SF-12, Health and Well-Being Questionnaire; NEMC, New England Medical Center.

^a^ Percentages based on predeployment N (visit 1) and may not sum to 100% due to missing data.

^b^ Very small but significant differences in age (F3 = 16.01; *P* < .001, ηp2 = .02), marital status (χ^2^_3_ = 48.20;* P* < .001; φ = .14), rank (χ^2^_6_ = 69.17; *P* < .001; φ = .16), and percentage of previously deployment Marines (N = 2,585; χ^2^_3_ = 1.17; *P* < .005; φ = .07) were detected between cohorts.

^c^ Percentages based on number of Marines who reported TBI at visit 1. Small but significant variations in rates of TBI were found in LOC and AMS with deployment history (N = 1,560; χ^2^_2_ = 6.12; *P* = .013; φ = .06).

^d^ Percentages based on number of Marines who reported TBI with LOC and AMS at visit 1.

Of the 1,562 (60.5%) Marines who reported prior head injury, 56.9% incurred at least 1 head injury with combined LOC and AMS symptoms. Small but significant variations in rates of TBI were found in LOC and AMS with deployment history; 54.1% of previously deployed Marines self-reported TBI with LOC and AMS compared with 60.4% of never-deployed Marines. Duration of unconsciousness did not vary significantly with deployment history.

Approximately half (51.3%) of Marines had at least 1 prior deployment at the time of enrollment in MRS. Previously deployed Marines accounted for 46.1% to 55.3% of each battalion; percentage differences were significant but small.

Mean (SD) PHC scores were slightly lower for previously deployed (53.27 [6.13]) compared with never-deployed Marines (54.17 [6.10]) (N = 2,514; F1 = 13.92; *P* < .001; ηp^2^ = .01). However, we found no deployment-related differences in age-adjusted MHC scores.

ICD-9-CM mental disorder diagnoses retrieved from the CHAMPS database showed that 193 (7.4%) of the 2,593 enrolled Marines had either 1 diagnosis (3.70%) or multiple (3.74%) diagnoses (Table 3). After controlling for time spent in the military before deployment, there were no significant differences in the number of mental health diagnoses per subject between previously deployed and never-deployed Marines. We did, however, find moderately significant relationships between deployment history and rates of diagnosed PTSD and diagnosed suicidal ideation. Of the 193 Marines with at least 1 ICD-9-CM diagnosis, 133 (68.9%) were previously deployed and 60 (31.1%) were never deployed. Approximately 19.6% of previously deployed Marines with an ICD-9-CM diagnosis had PTSD, compared with only 1.7% of never-deployed Marines. Conversely, only 6.0% of previously deployed Marines with an ICD-9 diagnosis were seen for suicidal ideation, compared with 21.7% of never-deployed Marines.

**Table 3:** Mental Health Diagnoses of Marines in the Marine Resiliency Study at Predeployment (T1) Assessment^a^

Diagnosis	Battalion 1	Battalion 2	Battalion 3	Battalion 4	Total

	n (%) (n = 315)	n (%) (n = 721)	n (%) (n = 671)	n (%) (n = 886)	n (%) (N = 2,593)
**Substance-related disorders**									
Alcohol	4 (1.27)	45 (6.24)	28 (4.17)	39 (4.40)	116 (4.47)
Drug	1 (0.32)	11 (1.53)	6 (0.89)	0	18 (0.69)
Adjustment disorders	6 (1.90)	26 (3.61)	27 (4.02)	21 (2.37)	80 (3.09)
**Mood disorders**									
Major depression	2 (0.63)	7 (0.97)	4 (0.60)	4 (0.45)	17 (0.66)
Bipolar disorder	0	1 (0.14)	0	0	1 (0.04)
Dysthymia	0	5 (0.69)	3 (0.45)	4 (0.45)	12 (0.46)
Depression, not otherwise specified	2 (0.63)	11 (1.53)	7 (1.04)	8 (0.90)	28 (1.08)
Mood disorder, not otherwise specified	2 (0.63)	0	0	0	2 (0.08)
Personality disorders	0	8 (1.11)	5 (0.75)	7 (0.79)	20 (0.77)
**Psychotic disorders**									
Schizophrenia	0	0	0	0	0
Brief psychotic disorder	0	0	0	0	0
Psychosis, not otherwise specified	0	1 (0.14)	0	0	1 (0.04)
**Anxiety disorders**								
Panic disorder	1 (0.32)	5 (0.69)	1 (0.15)	3 (0.34)	10 (0.39)
Generalized anxiety disorder	1 (0.32)	0	1 (0.15)	1 (0.11)	3 (0.12)
Obsessive-compulsive disorder	0	0	0	0	0
Phobias	0	1 (0.14)	0	0	1 (0.04)
Acute stress	0	3 (0.42)	1 (0.15)	1 (0.11)	5 (0.19)
Posttraumatic stress disorder^b^	2 (0.63)	10 (1.39)	8 (1.19)	8 (0.90)	28 (1.08)
Anxiety, not otherwise specified	2 (0.63)	9 (1.25)	8 (1.19)	9 (1.02)	28 (1.08)
**Somatoform/dissociative/factitious disorders**									
Dissociative disorder	0	0	0	0	0
Factitious disorder	0	0	0	0	0
Conversion disorder	0	1 (0.14)	0	1 (0.11)	2 (0.08)
Somatoform disorders	0	0	0	0	0
**Suicidal ideation**								
Ideation^c^	0	4 (0.55)	5 (0.75)	4 (0.45)	13 (0.50)
Ideation and attempt	1 (0.32)	4 (0.55)	2 (0.30)	1 (0.11)	8 (0.31)
**Other mental disorders**								
Organic conditions	0	0	1 (0.15)	0	1 (0.04)
Eating disorder	0	0	0	0	0
Unspecified mental disorder	1 (0.32)	0	0	0	1 (0.04)
Psychological factors, physical condition	0	1 (0.14)	2 (0.30)	0	3 (0.12)
Sleep disorder	0	2 (0.28)	7 (1.04)	5 (0.56)	14 (0.54)
**All other**	2 (0.63)	13 (1.8)	11 (1.64)	8 (0.90)	34 (1.31)

^a^ Table reflects data derived from the Career History Archival Medial and Personnel System. Percentages based on the total N enrolled.

^b^ Rates of PTSD diagnosis were significantly influenced by deployment history (N = 193; χ^2^_1_ = 10.99; *P* < .001; φ = .24).

^c^ Rates of diagnosed suicidal ideation were significantly influenced by deployment history (N = 193; χ^2^_1_ = 10.45; *P* = .001; φ = −.23).

## Discussion

MRS Marines are exclusively male and, compared with all enlisted Marines from 2008 through 2010 (Navy and Marine Corps Public Health Center data), are younger, more often unmarried, and of lower rank, similar to the demographics of war-deploying battalions (23).

As expected, scores on the SF-12 measure of functional health at predeployment are similar to population norms. Recently published studies provide evidence that low SF-12 scores and predeployment mental health diagnoses can serve as markers of vulnerability (16,24). Larson et al (24) reported that 23% of Marines seen by an in-theater mental health provider had a prior ICD-9-CM mental health diagnosis; for service members with a prior diagnosis, the highest rates of rediagnosis were for attention deficit disorder (57%) and PTSD (55%). It is therefore conceivable that MRS participants would be more likely to need in-theater treatment. The broad scope and prospective design of MRS should enable us to test this assumption and to further incorporate additional psychosocial and biological measures to better understand factors predictive of relapse and resilience.

Certain features of the enrolled sample limit its generalizability. All participants are male members of either Marine Corps or Navy (primarily health care personnel attached to Marine units), so women, civilians, and members of other services are not represented. Also, few members of the reserves and no members of the National Guard are enrolled. On the other hand, because MRS cohorts are enrolled from among Marine Corps ground combat units preparing to deploy, our results should prove generalizable to the Marine Corps, whose exposure to potentially traumatic war zone events is second only to that of the Army, as indexed by cumulative casualty rates (25). The description of participant characteristics before deployment combined with future longitudinal data analysis may allow researchers to identify modifiable multisystem risk and resilience factors for combat-related PTSD.
