# Erratum, Vol. 9, May 10 Release

**DOI:** 10.5888/pcd9.110134e

**Published:** 2012-05-24

Because of an editing error, we inadvertently omitted the name of an author from the Author Affiliations list in the article “Predictors of Risk and Resilience for Posttraumatic Stress Disorder Among Ground Combat Marines: Methods of the Marine Resiliency Study.” Caroline M. Nievergelt, University of California-San Diego, La Jolla, California, should have been included. This correction was made to our website on May 11, 2012, and appears online at http://www.cdc.gov/pcd/issues/2012/11_0134.htm. We regret any confusion or inconvenience this error may have caused.

